# How plants cope with fast primary root elongation inhibition

**DOI:** 10.3389/fpls.2023.1187634

**Published:** 2023-05-31

**Authors:** Ziwen Qiu, Cheng Zeng, Huiming Deng, Zeping Shen, Huibin Han

**Affiliations:** College of Bioscience and Bioengineering, Jiangxi Agricultural University, Jiangxi, Nanchang, China

**Keywords:** auxin, TMK, RALF1, FERONIA, fast root growth inhibition

## TIR1/AFB and TMK1 act antagonistically in auxin-mediated fast primary root elongation inhibition

Plants adjust their growth rapidly in response to various stimuli, thus improving their fitness to ever-changing environments. In most cases, including plasma membrane (PM) depolarization, H^+^-pump activation, transient cytosolic Ca^2+^, and pH changes, are regulated by the plant phytohormone auxin (Friml, 2022). Moreover, auxin also induces fast (within 2 min) and reversible primary root elongation inhibition, which particularly requires the cytoplasm localized AFB1 receptor together with TIR1 and other AFBs, to operate in an unexpected non-transcriptional manner (Fendrych et al., 2018; Prigge et al., 2020; Dubey et al., 2021; Li et al., 2021; Serre et al., 2021; Chen et al., 2023; Dubey et al., 2023).

The influx auxin carrier AUX1 imports IAA^−^ into cells with two H^+^, and passive diffusion also delivers auxin into the cells. On one hand, TIR1/AFB receptors perceive auxin to promote cellular H^+^ influx across the PM through so far undiscovered channels or transporters/antiporters, resulting in rapid apoplast alkalinization. The Ca^2+^ transient contributes to H^+^ influx (Li et al., 2021; Li et al., 2022a); however, it remains elusive whether undefined Ca^2+^ channels are required for H^+^ influx. On the other hand, the auxin-TIR1/AFB module acts through CYCLIC NUCLEOTIDE-GATED CHANNEL 14 (CNGC14), a Ca^2+^ channel, to rapidly stimulate PM depolarization (Serre et al., 2021; Serre et al., 2022). As a consequence, primary root elongation is repressed rapidly (Fendrych et al., 2018; Li et al., 2021; Serre et al., 2021; Serre et al., 2022). Recently, it has been suggested that adenylate cyclase (AC) activity is essential for TIR1/AFB receptor function and auxin perception (Qi et al., 2022). Auxin stimulates the AC activity to produce cAMP, thus executing its long-term effect on primary root elongation. Notably, AC activity is not required for auxin-TIR1/AFB signaling-mediated fast primary root elongation inhibition (Qi et al., 2022). Notably, the involvement of cGMP signaling in the auxin-induced fast primary root elongation inhibition requires further investigation.

**Figure 1:**
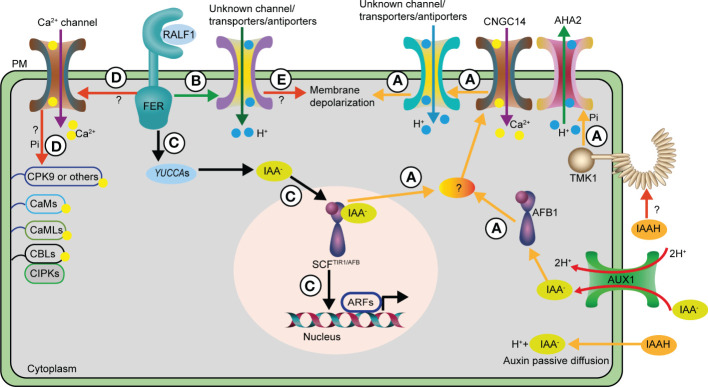
Auxin and RALF1 mediate rapid *Arabidopsis* primary root elongation inhibition via distinct routes. **(A)** The AUX1 auxin influx transporter and passive diffusions deliver auxin into the cells, then intracellular auxin is mainly perceived by cytoplasm AFB1 receptor, together with other SCF^TIR1/AFB^ receptors in the nucleus to trigger a rapid CNGC14-mediated Ca^2+^ influx through unknown factors. The Ca^2+^ transient contributes to the H^+^ influx into cells across the PM via so far unidentified channels or transporters/antiporters, which ultimately leads to apoplast alkalinization and PM depolarization. As a result, primary root elongation is inhibited rapidly. In contrast, cell surface localized TMK1 kinase recognizes auxin via an undefined manner to phosphorylate AHA2, a H+-ATPase, to pump H^+^ to apoplast, resulting in apoplast acidification and promotion of primary root elongation. Hence, the SCF^TIR1/AFB^ receptor and TMK1 kinase antagonize the auxin-dependent rapid primary root elongation inhibition. **(B)** RALF1 and its corresponding receptor FER may act through undefined channels or transporters/antiporters to trigger apoplast alkalinization, resulting in rapid and reversible primary root elongation inhibition. **(C)** The RALF1-FER module promotes auxin biosynthesis via increasing *YUCCA*s expression, thus inducing the canonical nucleus SCF^TIR1/AFB^ transcriptional pathway for its long-term effect on primary root elongation inhibition. **(D)** Unmapped Ca^2+^ channels and signaling pathways that may be involved in RALF1-FER mediated both rapid and sustained effect on primary root elongation inhibition. **(E)** RALF1 acts via FER receptor to regulate undefined channels or transporters/antiporters to trigger PM depolarization in an unknown manner for the regulation of rapid response in primary root. AHA2, H^+^-ATPase 2; ARF, AUXIN RESPONSE FACTOR; CNGC14, Cyclic NUCLEOTIDE-GATED CHANNEL 14; PM, plasma membrane; TIR1/AFB, TRANSPORT INHIBITOR RESPONSE1/AUXIN-SIGNALLING F-BOX PROTEIN; TMK1, TRANSMEMBRANE KINASE 1; AUX1, AUXIN RESISTANT 1; CaM, CALMODULIN; CMLs, CaM-LIKE PROTEINs; CDPKs, Ca^2+^-DEPENDNT PROTEIN KINASEs; CBLs, CALCINEURIN B-LIKE PROTEINs; CIPKs, CBL-INTERACTING PROTEIN KINASEs; CPK9, CALCIUM-DEPENDENT PROTEIN KINASE 9; Pi, phosphorylation.

In addition to TIR1/AFB perception machinery, apoplastic and PM-localized auxin signaling components have been proposed for years; AUXIN BINDING PROTEIN 1 (ABP1) and TRANSMEMBRANE KINASEs (TMKs) are excellent candidates (Cao et al., 2019; Lin et al., 2021; Friml et al., 2022). Remarkably, TMK1 transmits the auxin signal in an unraveled manner to phosphorylate AHA2, a H^+^-ATPase, at the well-known Thr947 activation site, resulting in apoplast acidification and promotion of primary root elongation (Li et al., 2021). Conversely, ABP1 has recently been reported to mediate auxin-induced fast (2 min) phospho-response together with TMK1 (Friml et al., 2022). However, the loss-of-function *abp1* mutant responds normally to auxin in fast primary root elongation inhibition (Li et al., 2021). Nonetheless, further analysis of *abp1 tmk1* double mutant in the fast auxin response is further needed (Rodriguez et al., 2022). Collectively, the TIR1/AFB and cell surface TMK1 kinase mediate antagonistic auxin signaling to fine-tune primary root elongation rapidly, but the mechanisms are still beyond our current understanding (**Figure 1**).

Besides apoplast alkalinization, other cellular processes such as cortical microtubule re-orientation (Adamowski et al., 2019) and vacuolar expansion (Dünser et al., 2019; Dünser et al., 2022) have been implicated in cell elongation. In elongating cells, the orientation of microtubules can either limit or allow cell expansion in a certain direction. Vacuoles are unique plant organelles, and their dimensions play essential roles in defining plant cell expansion rates. During cellular elongation, the size of the vacuole and its relative occupancy of the cell dramatically increase to 80%–90% of the cellular volume, which leads to rapid cellular growth (Dünser et al., 2019; Dünser et al., 2022). Therefore, microtubule re-orientation and vacuolar expansion may potentially be part of the machinery in rapid primary root elongation inhibition. Evidence has been shown that auxin does not rapidly induce microtubule re-orientation or vacuolar expansion (Li et al., 2021).

Ca^2+^ signaling consists of an array of receptors that perceive extracellular cues and Ca^2+^ channels that transport Ca^2+^ into the cells, formulating a specific Ca^2+^ signature (Tang et al., 2020). CALMODULIN (CaM), CaM-LIKE PROTEINs (CMLs), Ca^2+^-DEPENDNT PROTEIN KINASEs (CDPKs), and CALCINEURIN B-LIKE PROTEINs (CBLs) have been identified in land plants as Ca^2+^ sensors. Plant-specific CBL-INTERACTING PROTEIN KINASEs (CIPKs) act as major downstream signaling components of the CBL sensors to orchestrate central CBL-CIPK signaling networks and fine-tune plant adaptive growth in response to developmental and environmental cues (Tang et al., 2020). Auxin immediately triggers ion fluxes across the PM in root cells. In particular, transient increased cytosolic Ca^2+^ and increased apoplastic pH are detected less than 30 s after auxin treatment (Li et al., 2021; Serre et al., 2022). Therefore, the Ca^2+^ transient and external pH changes represent very early responses to auxin. Most likely, auxin acts via TIR1/AFB receptors to activate CNGC14 channel (Shih et al., 2015; Dindas et al., 2018; Li et al., 2021; Serre et al., 2022). The increased Ca^2+^ thus transiently initiates fast H^+^ influx and apoplast alkalinization. Consequently, primary root elongation is rapidly inhibited (**Figure 1**) (Li et al., 2021; Serre et al., 2022). How auxin regulates the well-known Ca^2+^ signaling pathways to mediate fast root elongation regulation (Tang et al., 2020), however, is largely unknown. On the other hand, it is unclear whether so far undiscovered channels or transporters/antiporters are involved in auxin-mediated fast H^+^ influx into the cells, as auxin induces the K^+^ efflux (Li et al., 2021; Li et al., 2022a).

Auxin rapidly arrests primary root elongation, which is too quick to be associated with transcriptional regulation, suggesting that this rapid elongation regulation may occur at the protein level (Fendrych et al., 2018; Li et al., 2021; Serre et al., 2021). Protein (de)phosphorylation is a prominent post-translational modification involved in many signaling pathways (Vu et al., 2018), and auxin has been reported to provoke a fast (2 min) protein phosphorylation through the receptors, TIR1/AFB, ABP1, and TMK1 (Han et al., 2021; Lin et al., 2021; Friml et al., 2022; Roosjen et al., 2022; Kuhn et al., 2022). Indeed, this fast protein phosphorylation analysis in root tips has successfully led to the identification of AHA2 that antagonizes TIR1/AFB-mediated rapid primary root elongation modulation (Han et al., 2021; Li et al., 2021). Additionally, a RAF-like protein kinase was identified as a central mediator of fast auxin-mediated phosphorylation, but its role in the fast responses of primary root needs further investigation in the future (Kuhn et al., 2022). Together with the fast phospho-response in the auxin receptor mutants (Lin et al., 2021; Friml et al., 2022; Kuhn et al., 2022; Roosjen et al., 2022), it will provide new access to identify unprecedented auxin-mediated machinery that enables plants to adjust primary root elongation rapidly through the non-canonical auxin signaling cascade. We also have to note that auxin may also trigger the rapid flow of K^+^ and other ions across the membrane (Ward et al., 2009; Li et al., 2021) and cell wall extensibility (Du et al., 2020), which will ultimately lead to the primary root elongation inhibition. A further investigation of ion exchange and cell wall plasticity in the auxin-mediated fast responses of primary root at the translational or post-translational level needs to be carried out.

## RALF1-FER module rapidly arrests primary root elongation

The small cysteine-rich polypeptide, RAPID ALKALINIZXATION FACTOR1 (RALF1), has also been shown to rapidly (within 2 min) arrest primary root elongation in a similar way to auxin (Li et al., 2021; Li et al., 2022b). The application of the synthetic RALF1 peptide may also generate a rapid net H^+^ influx across the PM into cells; nevertheless, this rapid net H^+^ influx and primary root elongation are abolished in FERONIA (FER) receptor mutant (Gjetting et al., 2020; Li et al., 2022b). Their result further suggests that the RALF1-FER module functions in a non-transcriptional manner to actuate rapid apoplast alkalinization and root elongation inhibition (Li et al., 2022b). It is implausible for RALF1 to directly regulate AHAs proton pump, as the tested *aha* mutants show normal responses to RALF1 peptide treatment within a time scale of 0 to 6 h, suggesting that the existence of so far undiscovered channels or transporters/antiporters is responsible for RALF1-inhibited rapid primary root elongation (Li et al., 2021; Li et al., 2022b). Unexpectedly, RALF1 and auxin independently suppress primary root elongation rapidly, supported by the fact that *fer* responds normally to short-term auxin treatment, and *tir* triple and *tmk* mutants respond normally to RALF1 peptide (Li et al., 2021; Li et al., 2022b). Notably, RALF1-FER modulates the biosynthesis of auxins via elevating the expression of auxin biosynthesis gene *YUCCA*s, and the accumulated auxin then inhibits primary root elongation acts through TIR1/AFB signaling in a long-term dimension (Li et al., 2022b). Thus, RALF1 exhibits both fast and sustained effects on primary root elongation through different mechanisms (**Figure 1**).

FER plays multiple roles in plant development, including cortical microtubule re-orientation (Malivert et al., 2021; Lin et al., 2022; Tang et al., 2022) and intracellular expansion of the vacuole (Dünser et al., 2019). However, whether microtubule re-orientation or vacuole expansion is involved in RALF1-FER module-mediated rapid response requires further investigation. Additionally, it is also necessary to examine whether RALF1-FER initiates rapid PM depolarization in parallel with apoplast alkalinization and, if so, what the underlying mechanisms are (**Figure 1**).

A fast RALF1-induced PM protein phosphorylation assay has led to the identification of CALCIUM-DEPENDENT PROTEIN KINASE 9 (CPK9) as a strong candidate to enhance cytoplasmic calcium concentrations (Haruta et al., 2014). NORTIA (NTA) functions as a Ca^2+^ sensor in FER-mediated pollen tube reception (Gao et al., 2022). Therefore, it is an open question to test the involvement of CNGC14, NTA, or other unmapped Ca^2+^ channels and signaling components (Tang et al., 2020) in RALF1-FER-dependent fast and sustained primary root elongation inhibition (**Figure 1**).

FER is a Ser/Thr receptor kinase that phosphorylates itself or substrates in a context-specific manner (Zhu et al., 2021). RALF1 induces FER phosphorylation within 5 min, indicating uncharacterized rapid protein phosphorylation networks to operate the rapid effect on primary root elongation through RALF1-FER signaling (Haruta et al., 2014). The newly developed timsTOF Pro mass spectrometer, powered by parallel accumulation serial fragmentation (PASEF) acquisition mode and trapped ion mobility spectrometry (TIMS) technology, paves the way to 4D-proteomics (Loginov et al., 2021). This approach will provide essential information on fast protein phosphorylation and will contribute to identify undefined robust protein networks involved in auxin or RALF1-FER module in both fast and sustained primary root elongation inhibition.

## Future perspectives

Unlike animals, plants cannot run when they meet environmental stressors. Rapidly adjusting their growth is one of the most effective strategies to avoid the negative effects of diverse stressors on development and growth. The disclosed fast (within 2 min) and reversible effects of auxin and RALF1 peptide provide mechanistic insights into rapid primary root elongation regulation. Nevertheless, open questions remain to be unanswered. The key unresolved question is how TIR1/AFB and TMK1 kinase confer primary root rapid reactions, and which downstream players function in non-canonical auxin actions. Phosphoproteomics analysis has revealed that many proteins are rapidly phosphorylated independently of the TIR1/AFB pathway (Han et al., 2021). Only a small portion of the identified proteins overlaps with auxin-mediated transcriptional regulation (Han et al., 2021; Friml et al., 2022; Kuhn et al., 2022; Roosjen et al., 2022). Therefore, an in-depth analysis of fast phosphorylation assays in related receptor mutants (Han et al., 2021; Friml et al., 2022; Kuhn et al., 2022; Roosjen et al., 2022) will help identify the downstream players that are responsible for auxin-mediated fast primary root elongation regulation. On the other hand, it would be appealing to uncover the kinase cascades that endow the rapidity of the TIR1/AFB-TMK-dependent non-canonical auxin signaling or the RALF1-FER dependent signaling, although only a few kinases have been confirmed in these fast signaling pathways (Haruta et al., 2014; Kubeš and Napier, 2019; Kuhn et al., 2022). We have to note that the rapid elongation inhibition of *Arabidopsis* primary root depends not only on the AFB1 subcellular localization but also on specific AFB1 protein properties (Prigge et al., 2020; Chen et al., 2023; Dubey et al., 2023). Furthermore, the auxin effect on endocytic PIN trafficking is also a rapid, non-transcriptional, and TIR1/AFB-independent biological process (Friml, 2022). Hence, auxin may also regulate its target protein dynamics through endocytic trafficking to execute its rapid inhibitory effect on primary root elongation.

RALF1 also regulates dynamics and partitioning of FER at PM via clathrin-mediated endocytosis (CME), and the *clc2-1/3-1* mutant, impaired in CME, shows resistance to RALF1 peptide in root growth inhibition (Yu et al., 2020). It is likely that RALF1 may trigger a rapid dynamic change of FER and FER-targeted PM proteins to initiate the early signals (such as pH, ROS, Ca^2+^) and MITOGEN-ACTIVATED PROTEIN KINASE (MAPK) cascades to exert its rapidness (Zhu et al., 2021), but this requires detailed investigation. Moreover, FER shows differential affinity to RALF peptides, and the involvement of other RALFs in this rapid primary root response remains unknown. The RALF1-FER signaling in the plant kingdom has been revealed, and RALFs and their (co)receptors co-evolved from bryophytes to seed plants (Zhu et al., 2021). Hence, it is intriguing to reveal that whether the RALF1-FER signaling in plant rapid primary root elongation is evolutionary conserved. Additionally, it is worth examining whether the FER interacting proteins (Du et al., 2016) are required for both fast and long-term effects of RALF1. Another crucial unanswered question is how RALF1 regulates FER kinase activity under different conditions or in diverse cell types, thus coordinating its biphasic effect on primary root elongation. Its potential players and the underlying mechanisms for the rapid activation or inactivation of RALF1-FER signaling remain largely unclear. It is also puzzling why plants utilize two independent pathways to rapidly inhibit primary root elongation via regulating H^+^ influx (**Figure 1**). Also, it is not clear at which physiological circumstances plants awaken RALF1-FER or auxin signaling, and how these independent pathways are integrated to adjust *Arabidopsis* primary root elongation rapidly. The answers to these fundamental but mysterious questions would assist in better elucidating the unprecedented auxin and RALF1-FER regulatory networks and provide a novel toolbox to improve agronomic traits.

## Author contributions

All authors listed have made a substantial, direct, and intellectual contribution to the work and approved it for publication.

## References

[B1] Adamowski M. Li L. Friml J. (2019). Reorientation of cortical microtubule arrays in the hypocotyl of arabidopsis thaliana is induced by the cell growth process and independent of auxin signaling. Int. J. Mol. Sci. 20, 3337. doi: 10.3390/ijms20133337 31284661 PMC6651120

[B2] Cao M. Chen R. Li P. Yu Y. Zheng R. Wang X.. (2019). TMK1-mediated auxin signalling regulates differential growth of the apical hook. Nature 568, 240–243. doi: 10.1038/s41586-019-1069-7 30944466

[B3] Chen H. Li L. Qi L. Friml J. (2023). Distinct functions of TIR1 and AFB1 receptors in auxin signalling. Preprint at bioRxiv. doi: 10.1101/2023.01.05.522749 37393433

[B4] Dindas J. Scherzer S. Roelfsema M. R. G. von Meyer K. Müller H. M. Al-Rasheid K. A. S.. (2018). AUX1-mediated root hair auxin influx governs SCF^TIR1/AFB^-type Ca^2+^ signaling. Nat. Commun. 9, 1174. doi: 10.1038/s41467-018-03582-5 29563504 PMC5862985

[B5] Du C. Li X. Chen J. Chen W. Li B. Li C.. (2016). Receptor kinase complex transmits RALF peptide signal to inhibit root growth in arabidopsis. Proc. Natl. Acad. Sci. U. S. A. 113, E8326–E8334. doi: 10.1073/pnas.1609626113 27930296 PMC5187724

[B6] Du M. Spalding E. P. Gray W. M. (2020). Rapid auxin-mediated cell expansion. Annu. Rev. Plant Biol. 71, 379–402. doi: 10.1146/annurev-arplant-073019-025907 32131604 PMC7733314

[B7] Dubey S. M. Han S. Stutzmanm N. Prigge M. J. Medvecká E. Platre M. P.. (2023). The AFB1 auxin receptor controls the cytoplasmic auxin response pathway in. Preprint at bioRxiv. doi: 10.1101/2023.01.04.522696 PMC10720607 37391902

[B8] Dubey S. M. Serre N. B. C. Oulehlová D. Vittal P. Fendrych M. (2021). No time for transcription-rapid auxin responses in plants. *Cold spring harb* . Perspect. Biol. 13, a039891. doi: 10.1101/cshperspect.a039891 PMC8327833 33648988

[B9] Dünser K. Gupta S. Herger A. Feraru M. I. Ringli C. Kleine-Vehn J. (2019). Extracellular matrix sensing by FERONIA and leucine-rich repeat extensins controls vacuolar expansion during cellular elongation in *Arabidopsis thaliana* . EMBO J. 38, e100353. doi: 10.15252/embj.2018100353 30850388 PMC6443208

[B10] Dünser K. Schöller M. Rößling A. K. Löfke C. Xiao N. Pařízková B.. (2022). Endocytic trafficking promotes vacuolar enlargements for fast cell expansion rates in plants. Elife 11, e75945. doi: 10.7554/eLife.75945 35686734 PMC9187339

[B11] Fendrych M. Akhmanova M. Merrin J. Glanc M. Hagihara S. Takahashi K.. (2018). Rapid and reversible root growth inhibition by TIR1 auxin signalling. Nat. Plants 4, 453–459. doi: 10.1038/s41477-018-0190-1 29942048 PMC6104345

[B12] Friml J. (2022). Fourteen stations of auxin. Cold Spring Harb. Perspect. Biol. 14, a039859. doi: 10.1101/cshperspect.a039859 34400554 PMC9159264

[B13] Friml J. Gallei M. Gelová Z. Johnson A. Mazur E. Monzer A.. (2022). ABP1-TMK auxin perception for global phosphorylation and auxin canalization. Nature 609, 575–581. doi: 10.1038/s41586-022-05187-x 36071161

[B14] Gao Q. Wang C. Xi Y. Shao Q. Li L. Luan S. A. (2022). Receptor-channel trio conducts Ca^2+^ signalling for pollen tube reception. Nature 607 (7919), 534–539. doi: 10.1038/s41586-022-04923-7 35794475 PMC9308748

[B15] Gjetting S. K. Mahmood K. Shabala L. Kristensen A. Shabala S. Palmgren M.. (2020). Evidence for multiple receptors mediating RALF-triggered Ca^2+^ signaling and proton pump inhibition. Plant J. 104, 433–446. doi: 10.1111/tpj.14935 32713048

[B16] Han H. Verstraeten I. Roosjen M. Mazur E. Rýdza N. Hajný J.. (2021). Rapid auxin-mediated phosphorylation of myosin regulates trafficking and polarity in arabidopsis. Preprint at bioRxiv. doi: 10.1101/2021.04.13.439603

[B17] Haruta M. Sabat G. Stecker K. Minkoff B. B. Sussman M. R. (2014). A peptide hormone and its receptor protein kinase regulate plant cell expansion. Science 343, 408–411. doi: 10.1126/science.1244454 24458638 PMC4672726

[B18] Kubeš M. Napier R. (2019). Non-canonical auxin signalling: fast and curious. J. Exp. Bot. 70, 2609–2614. doi: 10.1093/jxb/erz111 30854547 PMC6506764

[B19] Kuhn A. Roosjen M. Mutte S. Dubey S. M. Carrasco P. C. Monzer A.. (2022). A RAF-like kinase mediates a deeply conserved, ultra-rapid auxin response. Preprint at bioRxiv. doi: 10.1101/2022.11.25.517951 PMC10783624 38128538

[B20] Li L. Chen H. Alotaibi S. S. Pěnčík A. Adamowski M. Novák O.. (2022b). RALF1 peptide triggers biphasic root growth inhibition upstream of auxin biosynthesis. Proc. Natl. Acad. Sci. U. S. A. 119, e2121058119. doi: 10.1073/pnas.2121058119 35878023 PMC9351349

[B21] Li L. Gallei M. Friml J. (2022a). Bending to auxin: fast acid growth for tropisms. Trends Plant Sci. 27, 440–449. doi: 10.1016/j.tplants.2021.11.006 34848141

[B22] Li L. Verstraeten I. Roosjen M. Takahashi K. Rodriguez L. Merrin J.. (2021). Cell surface and intracellular auxin signalling for h^+^ fluxes in root growth. Nature 599, 273–277. doi: 10.1038/s41586-021-04037-6 34707283 PMC7612300

[B23] Lin W. Tang W. Pan X. Huang A. Gao X. Anderson C. T.. (2022). Arabidopsis pavement cell morphogenesis requires FERONIA binding to pectin for activation of ROP GTPase signaling. Curr. Biol. 32, 497–507. doi: 10.1016/j.cub.2021.11.030 34875229

[B24] Lin W. Zhou X. Tang W. Takahashi K. Pan X. Dai J. Ren H.. (2021). TMK-based cell-surface auxin signalling activates cell-wall acidification. Nature 599, 278–282. doi: 10.1038/s41586-021-03976-4 34707287 PMC8549421

[B25] Loginov D. S. Fiala J. Chmelik J. Brechlin P. Kruppa G. Novak P. (2021). Benefits of ion mobility separation and parallel accumulation-serial fragmentation technology on timsTOF pro for the needs of fast photochemical oxidation of protein analysis. ACS Omega 6, 10352–10361. doi: 10.1021/acsomega.1c00732 34056188 PMC8153767

[B26] Malivert A. Erguvan Ö. Chevallier A. Dehem A. Friaud R. Liu M.. (2021). FERONIA and microtubules independently contribute to mechanical integrity in the arabidopsis shoot. PloS Biol. 19, e3001454. doi: 10.1371/journal.pbio.3001454 34767544 PMC8612563

[B27] Prigge M. J. Platre M. Kadakia N. Zhang Y. Greenham K. Szutu W.. (2020). Genetic analysis of the arabidopsis TIR1/AFB auxin receptors reveals both overlapping and specialized functions. Elife 9, e54740. doi: 10.7554/eLife.54740.sa2 32067636 PMC7048394

[B28] Qi L. Kwiatkowski M. Chen H. Hoermayer L. Sinclair S. Zou M.. (2022). Adenylate cyclase activity of TIR1/AFB auxin receptors in plants. Nature 611, 133–138. doi: 10.1038/s41586-022-05369-7 36289340

[B29] Rodriguez L. Fiedler L. Zou M. Giannini C. Monzer A. Gelová Z.. (2022). Cell surface auxin signalling directly targets PIN-mediated auxin fluxes for adaptive plant development. Preprint at bioRxiv. doi: 10.1101/2022.11.30.518503

[B30] Roosjen M. Kuhn A. Mutte S. K. Boeren S. Krupar P. Koehorst J.. (2022). An ultra-fast, proteome-wide response to the plant hormone auxin. Preprint at bioRxiv. doi: 10.1101/2022.11.25.517949

[B31] Serre N. B. C. Kralík D. Yun P. Slouka Z. Shabala S. Fendrych M. (2021). AFB1 controls rapid auxin signalling through membrane depolarization in *Arabidopsis thaliana* root. Nat. Plants 7, 1229–1238. doi: 10.1038/s41477-021-00969-z 34282287 PMC7611683

[B32] Serre N. B. C. Wernerová D. Vittal P. Dubey S. M. Medvecká E. Jelínková A.. (2022). The AUX1-AFB1-CNGC14 module establishes longitudinal root surface pH profile. Preprint at bioRxiv. doi: 10.1101/2022.11.23.517700 PMC10414970 37449525

[B33] Shih H. W. DePew C. L. Miller N. D. Monshausen G. B. (2015). The cyclic nucleotide-gated channel CNGC14 regulates root gravitropism in *Arabidopsis thaliana* . Curr. Biol. 25, 3119–3125. doi: 10.1016/j.cub.2015.10.025 26752079

[B34] Tang W. Lin W. Zhou X. Guo J. Dang X. Li B.. (2022). Mechano-transduction via the pectin-FERONIA complex activates ROP6 GTPase signaling in arabidopsis pavement cell morphogenesis. Curr. Biol. 32, 508–517. doi: 10.1016/j.cub.2021.11.031 34875231

[B35] Tang R. J. Wang C. Li K. Luan S. (2020). The CBL-CIPK calcium signaling network: unified paradigm from 20 years of discoveries. Trends Plant Sci. 25, 604–617. doi: 10.1016/j.tplants.2020.01.009 32407699

[B36] Vu L. D. Gevaert K. De Smet I. (2018). Protein language: post-translational modifications talking to each other. Trends Plant Sci. 23, 1068–1080. doi: 10.1016/j.tplants.2018.09.004 30279071

[B37] Ward J. M. Mäser P. Schroeder J. I. (2009). Plant ion channels: gene families, physiology, and functional genomics analyses. Annu. Rev. Physiol. 71, 59–82. doi: 10.1146/annurev.physiol.010908.163204 18842100 PMC4790454

[B38] Yu M. Li R. Cui Y. Chen W. Li B. Zhang X.. (2020). The RALF1-FERONIA interaction modulates endocytosis to mediate control of root growth in arabidopsis. Development 147, dev189902. doi: 10.1242/dev.189902 32541006

[B39] Zhu S. Fu Q. Xu F. Zheng H. Yu F. (2021). New paradigms in cell adaptation: decades of discoveries on the CrRLK1L receptor kinase signalling network. New Phytol. 232, 1168–1183. doi: 10.1111/nph.17683 34424552

